# Prophylactic Dexmedetomidine Reduces Junctional Ectopic Tachycardia and Facilitates Postoperative Recovery in Pediatric Cardiac Surgery: A Systematic Review and Meta‐Analysis of Prospective Trials

**DOI:** 10.1002/pan.70182

**Published:** 2026-04-08

**Authors:** Michelle Singh, Arjun Anilkumar, Rohit Vondivillu Srinivasan, Jasim Abdul Jabbar, Naghul Malaichamy, Rethin Jayaraman

**Affiliations:** ^1^ First Faculty of Medicine Charles University Prague Czech Republic

## Abstract

**Objective:**

To evaluate the efficacy and safety of prophylactic dexmedetomidine in preventing Junctional Ectopic Tachycardia (JET) and its impact on postoperative recovery in pediatric congenital heart surgery, restricting analysis to prospective trials.

**Methods:**

We systematically searched PubMed, Scopus, and CENTRAL through October 16, 2025, for prospective randomized and quasi‐randomized trials. Retrospective cohorts were excluded. The primary outcome was postoperative JET incidence. Secondary outcomes included mechanical ventilation duration, ICU length of stay (LOS), Vasoactive‐Inotropic Score (VIS), and safety. Data were synthesized using random‐effects models and certainty of evidence was assessed using the GRADE framework.

**Results:**

Five prospective trials (*n* = 639) met the inclusion criteria. Prophylactic dexmedetomidine was associated with a significant reduction in postoperative JET incidence (OR 0.37; 95% CI 0.23–0.58; *p* < 0.0001; *I* ^2^ = 0%), supported by moderate‐certainty evidence. For secondary outcomes, pooled analyses suggested reductions in mechanical ventilation (MD −4.80 h) and ICU LOS (MD −19.83 h), but these were characterized by substantial clinical heterogeneity and low to very‐low certainty of evidence. A significant reduction in VIS emerged only in the sensitivity analysis; these findings remain hypothesis‐generating. No significant differences were observed for mortality or hypotension.

**Conclusions:**

In prospective pediatric cardiac surgery trials, prophylactic dexmedetomidine was associated with reduced postoperative JET, supported by moderate‐certainty evidence. While dexmedetomidine may help facilitate earlier recovery, its impact on secondary outcomes remains suggestive rather than definitive due to low evidence certainty. Future large‐scale, multicenter randomized trials are required to confirm if these potential benefits translate into consistent clinical improvements.

## 1 Introduction

The surgical stress response following cardiopulmonary bypass (CPB) triggers a surge in endogenous catecholamines that can precipitate Junctional Ectopic Tachycardia (JET), a postoperative arrhythmia delaying recovery [1, 2]. JET often necessitates prolonged sedation, increased vasoactive support, and extended mechanical ventilation to maintain hemodynamic stability, making its prevention an important perioperative objective [3, 4].

Intraoperative factors—including depth of anesthesia, sympathetic stimulation during sternotomy, and sedative choice—may influence postoperative arrhythmogenic risk [2]. Dexmedetomidine is a physiologically plausible adjunct in this setting. As a highly selective α2‐adrenergic agonist, it produces central sympatholysis by reducing sympathetic outflow and circulating catecholamines while stabilizing AV nodal conduction [5, 6, 7]. Unlike traditional antiarrhythmics, dexmedetomidine may provide arrhythmia prophylaxis alongside opioid‐sparing sedation without significant myocardial depression [8, 9, 10].

Although several studies have explored this association, the current evidence base is limited by variability in study design. Prior reviews combined retrospective and prospective data, introducing potential selection bias that may confound estimates of secondary recovery outcomes such as ventilation duration and ICU length of stay [3, 11]. In addition, optimal dosing strategies remain uncertain [11].

Therefore, an updated meta‐analysis is warranted. Unlike prior analyses, this study includes only prospective randomized and quasi‐randomized trials to provide a more reliable estimate of pharmacologic efficacy while minimizing confounding. The GRADE framework was used to assess the certainty of evidence. Our aim was to evaluate the efficacy of prophylactic dexmedetomidine in preventing JET and explore its potential effects on postoperative recovery outcomes, recognizing that these secondary findings remain hypothesis‐generating due to clinical heterogeneity.

## 2 Methods

This systematic review and meta‐analysis was conducted in accordance with the PRISMA 2020 statement (Table S1) [12]. The protocol was registered with PROSPERO (CRD420251170598).

### 2.1 Eligibility Criteria

Eligibility criteria were defined using the PICOS framework:

Population—children (≤ 18 years) undergoing congenital heart surgery with cardiopulmonary bypass (CPB).

Intervention—prophylactic dexmedetomidine administered intraoperatively or immediately postoperatively as part of the anesthetic or sedation regimen.

Comparison—placebo or standard care without dexmedetomidine.

Outcomes—primary: incidence of Junctional Ectopic Tachycardia (JET); secondary: mechanical ventilation duration, ICU length of stay, Vasoactive‐Inotropic Score (VIS), and safety outcomes including mortality and hypotension.

Study design—prospective randomized and quasi‐randomized controlled trials.

Given the scarcity of large‐scale, multicenter RCTs in pediatric cardiac anesthesia, quasi‐randomized trials were included to reflect the available prospective evidence. However, we acknowledge that pooling these with true RCTs represents a methodological compromise that may weaken the overall strength of inference and increase the risk of selection bias, as nonrandom allocation can artificially inflate effect estimates in perioperative research.

To ensure high internal validity, retrospective/observational studies were excluded to minimize unmeasured confounders such as survivorship and selection bias [3, 11]. Trials in which dexmedetomidine was initiated only after arrhythmia onset (therapeutic use) were also excluded. Eligible studies were required to prospectively assess postoperative JET during the early postoperative period, typically from the immediate post‐CPB phase through early ICU stay, most commonly within 8–72 h postoperatively [1, 2].

### 2.2 Information Sources and Search Strategy

We queried PubMed, Scopus, and the Cochrane Central Register of Controlled Trials (CENTRAL) from inception to October 16, 2025. To capture the full breadth of perioperative literature, we screened ClinicalTrials.gov and reference lists of relevant prior reviews [11]. The search strategy combined Medical Subject Headings (MeSH) and keywords for “Dexmedetomidine,” “Pediatric Cardiac Surgery,” and “Junctional Ectopic Tachycardia”. The full search strategies for all databases are provided in Table S2.

### 2.3 Selection Process

Search results were imported into Rayyan (Qatar Computing Research Institute). Two independent reviewers screened titles and abstracts for relevance to pediatric anesthesia and cardiac critical care. Full‐text articles were subsequently retrieved and assessed against eligibility criteria in duplicate. Disagreements were resolved through consensus or by a third reviewer's adjudication. A list of excluded studies and the specific reasons for their exclusion are detailed in Table S3.

### 2.4 Data Collection Process

Data extraction was performed in duplicate using a standardized, pilot‐tested form. Extracted variables included: trial characteristics (study design, blinding, funding, and diagnostic methods for JET detection, e.g., surface ECG vs. atrial wire electrograms); population characteristics (age, weight, and cardiac defects such as Tetralogy of Fallot); anesthetic protocols (dexmedetomidine loading/maintenance doses, timing relative to CPB, and control group sedation); and outcomes (JET definitions, event counts, and continuous data reported as mean ± SD).

When means and standard deviations were unavailable, they were estimated from medians and interquartile ranges using established methods [13, 14]. Blinding varied across trials: Rajput et al. [15] and Hassan et al. [16] were double‐blinded; El Amrousy et al. [17] blinded the care team and outcome assessors but not the administering anesthesiologist; Kadam et al. [18] and Wadile et al. [8] did not report blinding. VIS values were extracted as reported without recalculation, noting minor differences in formulae across studies.

### 2.5 Risk of Bias Assessment

Methodological quality was appraised in duplicate using the Cochrane Risk of Bias 2 (RoB 2) tool. We specifically assessed bias arising from the randomization process (including quasi‐random allocation), deviations from intended interventions (crucial for unblinded anesthetic trials), missing outcome data, measurement of the outcome (addressing JET detection heterogeneity), and selection of reported results.

### 2.6 Synthesis Methods

Meta‐analysis was conducted using Review Manager (RevMan 5.4). For dichotomous outcomes (JET, adverse events), Odds Ratios (OR) were calculated as the effect measure. For continuous outcomes (ICU stay, ventilation time, VIS), Mean Differences (MD) were used. A random‐effects model (inverse‐variance) was utilized for all analyses to account for expected clinical variability in surgical complexity and anesthetic protocols. Heterogeneity was quantified using the *I* ^2^ statistic. Sensitivity analyses were performed to assess the robustness of pooled estimates and to explore sources of heterogeneity, though these are interpreted as hypothesis‐generating. The reporting of these synthesis results aligns with the PRISMA 2020 checklist provided in Table S1.

### 2.7 Certainty of Evidence

The certainty of evidence for each outcome was graded using the GRADE approach. Certainty was assessed across domains of risk of bias, inconsistency, indirectness, imprecision, and publication bias. Outcomes were downgraded due to residual risks of bias, including quasi‐random allocation, unclear allocation concealment, and variability in JET monitoring approaches. The complete GRADE Summary of Findings, including detailed justifications for all certainty ratings, is presented in Table S4.

## 3 Results

### 3.1 Study Selection

**FIGURE 1:**
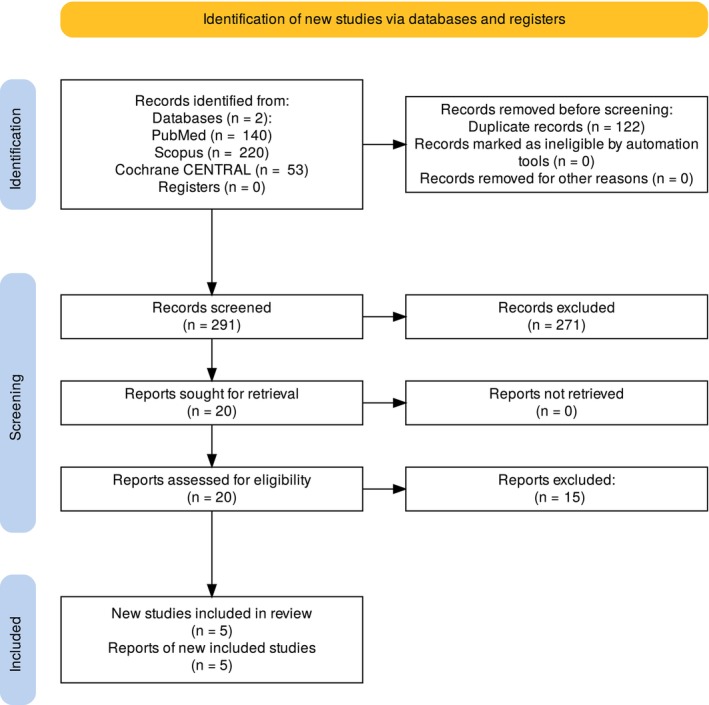
PRISMA flow diagram detailing the study selection process.

The literature search identified 413 records. After 122 duplicates were removed, 291 titles and abstracts were screened, and 267 were excluded. A total of 24 full‐text articles were assessed for eligibility, of which 19 were excluded. Ultimately, 5 studies met the full inclusion criteria and were included in the systematic review and quantitative meta‐analysis (Figure 1).

### 3.2 Study Characteristics

The 5 included studies were all prospective clinical trials (three randomized controlled trials and two quasi‐randomized trials) published between 2014 and 2024 [8, 15, 16, 17, 18]. The studies were conducted in India and Egypt. A total of 639 children undergoing congenital heart surgery were included, with 320 in the dexmedetomidine group and 319 in the control group. Specific dosing and infusion protocols are detailed in Table 1A, with key characteristics, including participant demographics and outcome definitions, summarized in Table 1B.

**TABLE 1A:** Dexmedetomidine dosing and infusion characteristics of included studies.

Study	Loading dose	Continuous infusion dose	Duration of continuous infusion	Mention of use of antiarrhythmic drug	JET monitoring duration	Infusion in the control group instead of dex	JET monitoring tool	Diagnostic criteria
Rajput et al. [14]	0.5 μg/kg	0.5 μg/kg/h	Discontinuation of mechanical ventilation, or 8 h	No	8 h post‐ CPB/ICU transfer	Normal saline	Surface ECG and Telemetry	Clinical diagnosis via surface ECG
Kadam et al. [18]	1 μg/kg	0.75 μg/kg/h	48 h	No	48 h post‐op	Fentanyl	Surface ECG	Clinical diagnosis via surface ECG
El Amrousy et al. [17]	0.5 μg/kg	0.5 μg/kg/h	48 h	No	48 h post‐op	Normal saline	Surface ECG and telemetry	Clinical diagnosis via surface ECG
Wadile et al. [8]	—	0.2–0.5 μg/kg/h	48 h	Yes	48 h post‐op	—	Atrial wire electrograms	Confirmation of AV dissociation (Gold Standard)
Hassan and El Haddad [16]	0.5 μg/kg	0.5 μg/kg/h	72 h	Yes	72 h post‐op	Normal saline	Atrial wire electrograms	Confirmation of AV dissociation (Gold Standard)

**TABLE 1B:** Overview and characteristics of included studies.

Study	Study type	Randomization process	Patients [control/dex (total)]	Age (mean)	Weight (kg) (control/dex)	Types of CHD (%)	Incidence of JET % (control/dex)	Timing of dexmedetomidine administration
Rajput et al. [15]	Prospective randomized controlled	By drawing of a chit by the operating room assistant	110/110 (220)	Control: 32.4 ± 17 months Dex: 32.5 ± 18 months	Control: 10.62 ± 4.36/Dex: 10.0 ± 4.12	TOF (100%)	20/9.1	Loading dose (0.5 μg/kg) + Intraoperative infusion
Kadam et al. [18]	Prospective quasi‐ randomized controlled	By alternating group assignment	47/47 (94)	Control: 161 ± 121 weeks/Dex: 152 ± 250 weeks	Control: 10.19 ± 6.74/Dex: 11.69 ± 10.96	TOF (100%)	23.4/8.5	Loading dose (1 μg/kg) + Intraoperative infusion
El Amrousy et al. [17]	Prospective randomized controlled	Sealed opaque envelopes	30/60 (90)	Control: 18.3 ± 5.4 months dex: 17.3 ± 4.1 months	Control: 12.6 ± 1.7/Dex: 12.4 ± 1.1	VSD (29%), TOF (12%), AVCD (16.6%), others	16.7/3.3	Loading dose (0.5 μg/kg) + Intraoperative infusion
Wadile et al. [8]	Prospective Quasi randomized controlled clinical trial	Alternate sampling method	85/70 (225)	Median: 9 months (Range: 2 days–144 months)	Median: 6.3 kg (Range: 1.8–38 kg)	VSD (42%), TOF (23%), others	24.7/14.2	Intraoperative infusion (No loading dose)
Hassan and El Haddad [16]	Prospective randomized double‐blind controlled trial	Computer‐generated sequence	40/40 (120)	Median: 15 months (Range 6–60)/Median: 18 months	Median: 9.5 kg (Range 5–30)/Median: 10.0 kg	VSD: (57.5%) ASD: (40%) AVSD: (2.5%)	30/10	Loading dose (0.5 μg/kg) + Intraoperative infusion

### 3.3 Risk of Bias in Included Studies

Risk of bias was appraised using the Cochrane RoB 2 tool (Table 2), with a summary of judgments across five domains presented in the traffic light plot (Figure S1). Methodological quality was mixed:

- Low Risk of Bias: One trial [16].
- Some Concerns: Two studies [15, 17] due to insufficient detail on randomization and allocation concealment
- High Risk of Bias: Two trials [8, 18] due to non‐random or poorly described sequence generation (quasi‐randomized; alternate sampling methods).

**TABLE 2:** Risk of bias assessment for included trials (RoB2).

Study	D1: randomization	D2: deviations from intervention	D3: missing outcome data	D4: measurement of outcome	D5: selection of reported result	Overall risk of bias
Rajput et al. [15]	Some concerns	Some concerns	Low risk	Low risk	Some concerns	Some concerns
Kadam et al. [18]	High risk	High risk	Some concerns	Low risk	Some concerns	High risk
El Amrousy et al. [17]	Some concerns	Low risk	Low risk	Low risk	Some concerns	Some concerns
Wadile et al. [8]	High risk	High risk	Some concerns	Low risk	Some concerns	High risk
Hassan and El Haddad [16]	Low risk	Low Risk	Low isk	Low risk	Low risk	Low risk

### 3.4 Synthesis of Results

#### 3.4.1 Primary Outcome: Incidence of Junctional Ectopic Tachycardia (JET)

**FIGURE 2:**
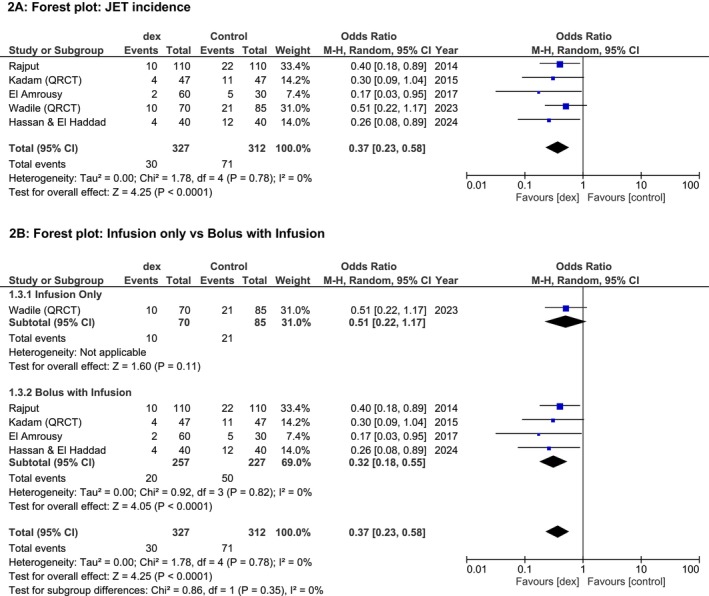
(A) Forest plot: JET incidence. (B) Forest plot: infusion only vs. bolus with infusion.

All five trials (*n* = 639) reported on the incidence of JET. The pooled analysis showed that prophylactic dexmedetomidine was associated with a significant 63% reduction in the odds of developing postoperative JET, though we acknowledge that including quasi‐randomized trials may introduce allocation bias (OR = 0.37; 95% CI 0.23 to 0.58; *p* < 0.0001; *I* ^2^ = 0%) [8, 15, 16, 17, 18]. (Figure 2).

To assess the impact of study quality, a sensitivity analysis excluding the two quasi‐randomized studies (Kadam et al. Wadile et al.) [8, 18] confirmed the primary finding. The pooled estimate remained significant and consistent (OR 0.32; 95% CI 0.17–0.60; *I* ^2^ = 0%), suggesting that the primary finding is robust and not disproportionately driven by studies at a higher risk of selection bias. (Figure S2).

A subgroup analysis (Figure 2B) demonstrated a statistically significant reduction in JET incidence for the bolus‐plus‐infusion cohort (OR = 0.32; 95% CI 0.18 to 0.55; *p* < 0.0001). The single trial utilizing an infusion‐only protocol (Wadile et al.) reported a result that was statistically insignificant (OR = 0.51; 95% CI 0.22 to 1.17; *p* = 0.11). However, due to the availability of only a single study in the infusion‐only subgroup and because no direct head‐to‐head comparison was performed between these strategies, this analysis remains strictly exploratory and hypothesis‐generating [8].

#### 3.4.2 Secondary Outcomes

**FIGURE 3:**
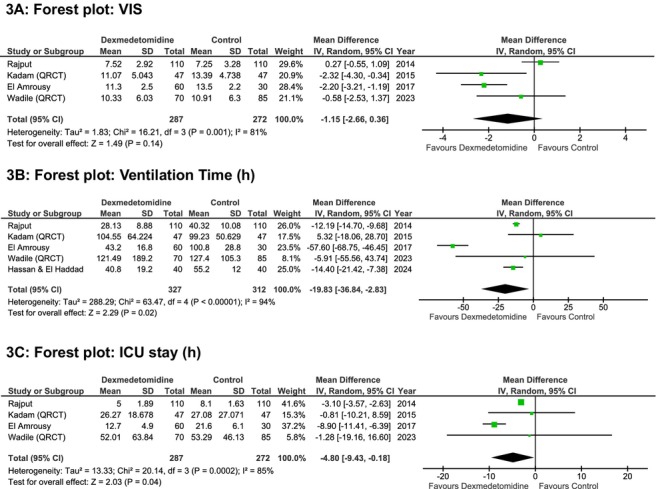
(A) Forest plot: VIS. (B) Forest plot: ventilation time. (C) Forest plot: ICU stay.

*Vasoactive‐Inotropic Score (VIS)*: Four studies (*n* = 519) reported VIS. The primary pooled analysis was not statistically significant (MD = −1.15; 95% CI –2.66 to 0.36; *p* = 0.14, *I* ^2^ = 81%) (Figure 3). A significant, homogenous effect was identified only in sensitivity analysis excluding two studies with inconsistent formulae (Rajput et al. and Wadile et al.) [8, 15]. (MD = −2.22; 95% CI –3.12 to −1.33; *p* < 0.00001; *I* ^2^ = 0%) (Figure S3). Given the dependence on sensitivity modeling and low certainty of underlying data, this finding is considered exploratory.

*Ventilation Time*: Four studies (*n* = 559) reported a significant reduction in the primary pooled analysis (MD = −4.8 h; 95% CI, −9.43 to −0.18; *p* = 0.04), but with substantial heterogeneity (*I* ^2^ = 85%) (Figure 3B). A post hoc sensitivity analysis excluding TOF‐only cohorts (Rajput et al. Kadam et al.) [15, 18] resolved heterogeneity (MD = −8.75 h; 95% CI –11.24 to −6.27; *p* < 0.00001; *I* ^2^ = 0%) (Figure S4). We attributed this to their specific clinical characteristics and smaller effect sizes which may serve as drivers of heterogeneity. We acknowledge that prioritizing this result may introduce post hoc selection bias and thus the overall evidence remains suggestive rather than definitive.

*ICU Stay*: Five studies (*n* = 639) reported ICU length of stay (MD = −19.83 h; 95% CI –36.84 to −2.83; *p* = 0.02), but with extreme heterogeneity (*I* ^2^ = 94%) (Figure 3C). We identified El Amrousy et al. (2017) [17] as a significant outlier due to its small, high‐variance control group and a drastically larger reported reduction in stay compared to the other included trials (−57.6 h). Excluding this study resulted in a significant, homogenous benefit (MD = −12.25 h; 95% CI –14.6 to −9.90; *p* < 0.00001; *I* ^2^ = 0%) (Figure S5). We acknowledge that relying on such exclusions may introduce post hoc selection bias. Consequently, consistent with our GRADE assessment, this recovery benefit remains inconclusive.

**FIGURE 4:**
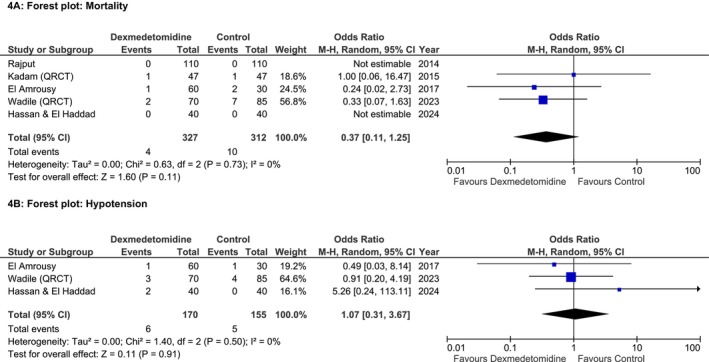
(A) Forest plot: mortality. (B) Forest plot: hypotension.

*Adverse Events*: There was no significant difference in the odds of mortality (OR = 0.37; 95% CI 0.11 to 1.25; *p* = 0.11; *I* ^2^ = 0%) (Figure 4) or hypotension (OR = 1.07; 95% CI 0.31 to 3.67; *p* = 0.91; *I* ^2^ = 0%) (Figure 4B).

### 3.5 Reporting Bias

A formal assessment of publication bias using a funnel plot was not performed due to the number of included studies (*n* = 5). Given the small number of trials and the predominance of single‐center studies, publication bias cannot be excluded.

### 3.6 Certainty of Evidence (GRADE)

Using the GRADE approach (Table S4), certainty for the primary outcome (JET) was rated as Moderate, downgraded due to risk of bias. Secondary outcomes were rated Low or Very Low due to a combination of bias, serious imprecision (wide confidence intervals/low event rates), and substantial heterogeneity.

Evidence was downgraded primarily due to selection and allocation bias from methodological weaknesses, such as the manual “chit” system used by an operating room assistant in the Rajput study [15], which lacks robust concealment, and the use of alternate allocation in quasi‐randomized trials [8,18]. Additionally, detection bias was identified due to varied diagnostic standards; while Wadile et al. [8] and Hassan et al. [16] used the “gold standard” of atrial wire electrograms to confirm AV dissociation, others relied on surface ECG/telemetry. This absence of electrophysiological confirmation may lead to misdiagnosis or an underestimation of JET incidence. Finally, the variability in monitoring windows, particularly the limited 8‐h window in the Rajput study [15], necessitated a more conservative assessment of overall certainty.

## 4 Discussion

### 4.1 Summary of Main Findings

This systematic review and meta‐analysis of five prospective trials suggests that prophylactic dexmedetomidine is associated with a clinically meaningful reduction in postoperative junctional ectopic tachycardia (JET) in pediatric cardiac surgery patients. Our analysis of 639 participants indicated a 63% reduction in the odds of developing JET (OR = 0.37; 95% CI 0.23–0.58; *p* < 0.0001) [8, 15, 16, 17, 18]. The primary finding demonstrated moderate‐certainty evidence with no statistical heterogeneity (*I* ^2^ = 0%) and remained consistent in analyses restricted to lower‐risk randomized trials, supporting its robustness. Secondary outcomes were less conclusive in unadjusted analyses.

While pooled analyses for ventilation time (MD = −4.8 h; *p* = 0.04) and ICU stay (MD = −19.83 h; *p* = 0.02) suggested a potential benefit, these findings showed high statistical heterogeneity (*I* ^2^ = 81%–94%) (Figure 3B,C) and low to very‐low certainty of evidence. The primary pooled analysis for vasoactive–inotropic score (VIS) was not statistically significant (*p* = 0.14), although a significant reduction emerged in sensitivity analyses. These exploratory analyses showed a consistent direction of effect but should be considered hypothesis‐generating rather than confirmatory. Importantly, no increase in adverse events was observed, with pooled estimates showing no significant difference in mortality (OR 0.37; *p* = 0.11) or hypotension (OR 1.07; *p* = 0.91) (Figure 4A,B) [8, 15, 16, 17, 18].

### 4.2 Comparison With Previous Literature

Our primary finding (OR 0.37) is numerically consistent with Ghimire et al. [11], but our methodology differs by restricting inclusion to prospective trials to minimize selection and survivorship bias. Retrospective cohorts are particularly prone to bias in recovery outcomes where sicker patients may be preferentially treated [3]. By excluding them, we aimed for a more conservative and internally valid estimate. However, the inclusion of quasi‐randomized trials remains a limitation. While sensitivity analyses (Figure S2) suggest the antiarrhythmic benefit is robust, the overall strength of inference is tempered by the risk of selection bias and variations in JET detection– specifically the lack of atrial wire monitoring in some studies, which may lead to an underestimation of incidence [15].

Finally, unlike previous reviews [11] that reported recovery benefits despite substantial inconsistency, our use of the GRADE framework identifies these secondary outcomes as inconclusive (Table S4). Consequently, while the reduction in JET is supported by moderate‐certainty evidence, recovery benefits remain supported by low to very‐low certainty due to unresolved heterogeneity. Our results for recovery outcomes should therefore be viewed as promising associations rather than definitive proof.

### 4.3 Mechanistic and Physiological Rationale

The antiarrhythmic effect of dexmedetomidine is mechanistically consistent with JET physiology. By blunting sympathetic surges during rewarming and early recovery, it reduces junctional automaticity and slows AV nodal conduction [4, 5, 6]. This offers a distinct advantage over amiodarone, which acts as a negative inotrope and vasodilator–risks that can exacerbate low cardiac output syndrome in a stunned post‐bypass heart [1, 8]. Our analysis explores this physiological benefit, though we acknowledge that while a significant reduction in Vasoactive‐Inotropic Score (VIS) emerged in the sensitivity analysis (MD −2.22; *p* < 0.00001; *I* ^2^ = 0%) (Figure S3), the primary pooled result remained non‐significant (*p* = 0.14).

While dexmedetomidine is a known sympatholytic, the potential reduction in Vasoactive Inotropic Score (VIS) in the dexmedetomidine group is likely mediated by improved hemodynamic stability secondary to effective arrhythmia prevention. Postoperative JET often causes significant hemodynamic deterioration, requiring compensatory increases in inotropic support to maintain cardiac output. By significantly reducing the incidence of JET, dexmedetomidine preserves atrioventricular synchrony and heart rate control, thereby minimizing the need to escalate exogenous catecholamine doses. Furthermore, its sedative and analgesic properties may reduce the endogenous stress response and sympathetic surges that often necessitate the titration of vasoactive agents in the early recovery phase [5, 6]. Notably, patients who remain in sinus rhythm rather than JET require less exogenous sympathomimetic support to maintain systemic perfusion.

Consequently, the observed improvements in recovery times are likely multifactorial: prevention of JET preserves cardiac output, while the sedative properties of dexmedetomidine reduce the need for respiratory‐depressant opioids [7]. We acknowledge the potential for covariance between the reduction in JET incidence and these secondary recovery outcomes. Postoperative JET is a known driver of hemodynamic instability, necessitating increased inotropic support and prolonged sedation, both of which consequently extend the time to extubation and ICU discharge [2]. Accordingly, the recovery benefits observed in the dexmedetomidine group may reflect indirect effects mediated through JET prevention, direct pharmacologic effects on sedation and sympathetic tone, or a combination of both, which cannot be disentangled at the aggregate level. This inherent interdependence contributes to the “Low” and “Very Low” GRADE certainty assigned to these outcomes, as the clinical benefits of rhythm stability and the drug's sedative profile are statistically and clinically intertwined.

### 4.4 Limitations

These findings should be interpreted with caution. Included trials were conducted in India and Egypt, potentially limiting generalizability to other healthcare systems. Differences in cardiopulmonary bypass strategies, perioperative temperature management, myocardial protection techniques, ICU staffing, sedation protocols, and extubation practices may influence both baseline incidence of Junctional Ectopic Tachycardia and recovery trajectories. While the sympatholytic and antiarrhythmic mechanisms of Dexmedetomidine are unlikely to be region‐specific, the magnitude of effect and downstream recovery benefits may vary across settings [8, 15, 16, 17, 18].

Second, the certainty of evidence differed across outcomes: the primary outcome was graded as moderate certainty, whereas secondary outcomes were rated low to very low due to substantial heterogeneity in pooled analyses of ventilation duration and ICU stay. Sensitivity analyses adjusting for clinical and methodological differences resolved this heterogeneity (*I* ^2^ = 0%), suggesting more consistent effects when these factors are accounted for; however, they remain exploratory. Third, inclusion of quasi‐randomized trials represents a limitation. Although included to capture available prospective evidence in a field with few large trials, pooling them with randomized studies may introduce selection bias and weaken overall inference. We accounted for this potential overestimation of benefit by downgrading the overall certainty of evidence for all outcomes within the GRADE framework (Table S4), while also employing a cautious interpretation of the pooled results, particularly for secondary outcomes where clinical heterogeneity was already substantial [8, 12, 18]. JET detection protocols were inconsistent across studies. Wadile et al. [8] and Hassan et al. [15] used atrial wire electrograms, the diagnostic gold standard, while others relied on surface ECG or telemetry [15, 17, 18], which may underestimate incidence or misdiagnose rhythms. Monitoring durations varied from 8 to 72 h, introducing potential detection bias, likely contributing to the ‘Moderate’ certainty of our primary outcome per GRADE [12, 15]. Neonates, the highest‐risk group, were underrepresented, which is a notable omission given they represent the highest‐risk population for postoperative arrhythmias. These findings should be interpreted cautiously and validated in larger, multinational cohorts.

### 4.5 Clinical Implications

Postoperative junctional ectopic tachycardia remains a frequent and clinically important complication following pediatric cardiac surgery, often necessitating deeper sedation, increased vasoactive support, and prolonged mechanical ventilation [1, 2]. In this context, strategies aimed at preventing JET rather than treating established arrhythmia are of particular relevance to perioperative anesthetic and critical care management [11].

This prospective trial‐only meta‐analysis suggests prophylactic dexmedetomidine may reduce postoperative JET, supported by moderate‐certainty evidence [8, 15, 16, 17, 18]. Its sympatholytic effects, minimal respiratory depression, and favorable hemodynamics make it a physiologically plausible preventive adjunct [4, 5, 7]. However, evidence on downstream outcomes—ventilation duration, ICU stay, and vasoactive needs—remains uncertain due to clinical heterogeneity and low‐to‐very‐low certainty. Routine use in ERAS protocols is not yet recommended and warrants evaluation in larger, well‐designed prospective trials.

Importantly, although exploratory subgroup analyses suggested a potential signal favoring bolus‐plus‐infusion regimens, current evidence is insufficient to define an optimal dosing strategy. Dexmedetomidine should be viewed as a potentially beneficial adjunct rather than a standardized prophylactic intervention, and its use should be individualized based on institutional protocols and patient characteristics until higher‐quality evidence becomes available.

### 4.6 Future Directions

The immediate priority is the conduction of large‐scale, multicenter RCTs with standardized definitions of JET, harmonized monitoring strategies, and predefined recovery outcomes. Trials should be designed to assess both arrhythmia prevention and recovery endpoints while accounting for mediation effects. Direct comparisons of dosing strategies and evaluation of long‐term safety outcomes are also warranted.

## 5 Conclusion

This systematic review and meta‐analysis of prospective trials suggests that prophylactic dexmedetomidine may reduce postoperative JET in pediatric cardiac surgery, although the strength of this inference is limited by the inclusion of quasi‐randomized trials and the moderate certainty of evidence [8, 15, 16, 17, 18]. The association remained consistent across studies and persisted on sensitivity analysis excluding quasi‐randomized trials.

In contrast, evidence for improvements in postoperative recovery outcomes—including mechanical ventilation duration, ICU length of stay, and vasoactive‐inotropic requirements remains inconclusive. Although sensitivity analyses suggested potential benefits, these findings were exploratory and limited by clinical heterogeneity and low to very‐low certainty of evidence. Current data is also insufficient to compare specific dosing strategies.

Overall, dexmedetomidine appears promising as a prophylactic strategy for reducing postoperative JET; however, its broader effects on recovery outcomes and optimal administration require confirmation in large, multicenter randomized controlled trials with standardized protocols, rigorous blinding, and harmonized outcome definitions [12].

## Author Contributions

Michelle Singh: conceptualization. Michelle Singh, Arjun Anilkumar, Rohit V. Srinivasan, Jasim Abdul Jabbar: methodology. Rohit V. Srinivasan, Michelle Singh, Arjun Anilkumar: data curation. Arjun Anilkumar: formal analysis/statistics. Michelle Singh, Rohit V. Srinivasan, Jasim Abdul Jabbar: investigation/screening. Michelle Singh, Rohit V. Srinivasan, Jasim Abdul Jabbar, Arjun Anilkumar: writing – original draft. Rohit V. Srinivasan, Arjun Anilkumar, Naghul Malaichamy, Rethin Jayaraman: writing – review and editing. Michelle Singh, Arjun Anilkumar, Rohit V. Srinivasan: supervision. Michelle Singh, Arjun Anilkumar, Rohit V. Srinivasan: project administration. All authors have read and approved the final manuscript.

## Funding

The authors have nothing to report.

## Ethics Statement

Ethical approval was not required for this study because it is a systematic review and meta‐analysis of previously published data, and no individual patient‐level information was collected or analyzed.

## Conflicts of Interest

The authors declare no conflicts of interest.

## Supporting information


**Table S1:** PRISMA 2020 checklist.
**Table S2:** Full search strategies.
**Table S3:** Excluded studies and reasons.
**Table S4:** GRADE summary of findings for prophylactic dexmedetomidine.
**Figure S1:** Traffic light plot.
**Figure S2:** Sensitivity analysis for incidence of JET. Sensitivity analysis for the primary outcome (Incidence of JET), excluding studies with a high risk of bias (Kadam et al. Wadile et al.).
**Figure S3:** Sensitivity analysis for vasoactive‐inotropic score (VIS). Sensitivity analysis for Vasoactive‐Inotropic Score (VIS), excluding studies with heterogeneous calculation methods (Rajput et al. 2014; Wadile et al. 2023).
**Figure S4:** Sensitivity analysis for ventilation time. Sensitivity analysis for ventilation time, excluding studies with clinically heterogeneous populations (TOF‐only cohorts) (Rajput et al. 2014; Kadam et al. 2015).
**Figure S5:** Sensitivity analysis for ICU stay. Sensitivity analysis for ICU stay, excluding statistical outlier (El Amrousy et al. 2017).

## Data Availability

All data used in this review are included within the article and Supporting Information.
