# Supplementary material for: Prophylactic Dexmedetomidine Reduces Junctional Ectopic Tachycardia and Facilitates Postoperative Recovery in Pediatric Cardiac Surgery: A Systematic Review and Meta‐Analysis of Prospective Trials
Source: Paediatr Anaesth. 2026 Apr 8;36(7):767–77. doi: 10.1002/pan.70182 (PMC13247623; doi:10.1002/pan.70182)
Supplement: Supplementary file 1 — Table S1: PRISMA 2020 checklist. Table S2: Full search strategies. Table S3: Excluded studies and reasons. Table S4: GRADE summary of findings for prophylactic dexmedetomidine. Figure S1: Traffic light plot. Figure S2: Sensitivity analysis for incidence of JET. Sensitivity analysis for the primary outcome (Incidence of JET), excluding studies with a high risk of bias (Kadam et al. Wadile et al.). Figure S3: Sensitivity analysis for vasoactive‐inotropic score (VIS). Sensitivity analysis for Vasoactive‐Inotropic Score (VIS), excluding studies with heterogeneous calculation methods (Rajput et al. 2014; Wadile et al. 2023). Figure S4: Sensitivity analysis for ventilation time. Sensitivity analysis for ventilation time, excluding studies with clinically heterogeneous populations (TOF‐only cohorts) (Rajput et al. 2014; Kadam et al. 2015). Figure S5: Sensitivity analysis for ICU stay. Sensitivity analysis for ICU stay, excluding statistical outlier (El Amrousy et al. 2017). [file PAN-36-767-s001.docx]

# **Supplementary Appendix**

### **Supplementary Table 1. PRISMA 2020 Checklist**

| **Section and Topic** | **Item #** | **Checklist item** | **Location where item is reported** |
| --- | --- | --- | --- |
| **TITLE** | | |  |
| Title | 1 | Identify the report as a systematic review. | 1 |
| **ABSTRACT** | | |  |
| Abstract | 2 | See the PRISMA 2020 for Abstracts checklist. | 3 |
| **INTRODUCTION** | | |  |
| Rationale | 3 | Describe the rationale for the review in the context of existing knowledge. | 4-5 |
| Objectives | 4 | Provide an explicit statement of the objective(s) or question(s) the review addresses. | 5 |
| **METHODS** | | |  |
| Eligibility criteria | 5 | Specify the inclusion and exclusion criteria for the review and how studies were grouped for the syntheses. | 5-6 |
| Information sources | 6 | Specify all databases, registers, websites, organisations, reference lists and other sources searched or consulted to identify studies. Specify the date when each source was last searched or consulted. | 6 |
| Search strategy | 7 | Present the full search strategies for all databases, registers and websites, including any filters and limits used. | 6 |
| Selection process | 8 | Specify the methods used to decide whether a study met the inclusion criteria of the review, including how many reviewers screened each record and each report retrieved, whether they worked independently, and if applicable, details of automation tools used in the process. | 6-7 |
| Data collection process | 9 | Specify the methods used to collect data from reports, including how many reviewers collected data from each report, whether they worked independently, any processes for obtaining or confirming data from study investigators, and if applicable, details of automation tools used in the process. | 7 |
| Data items | 10a | List and define all outcomes for which data were sought. Specify whether all results that were compatible with each outcome domain in each study were sought (e.g. for all measures, time points, analyses), and if not, the methods used to decide which results to collect. | 7 |
|  | 10b | List and define all other variables for which data were sought (e.g. participant and intervention characteristics, funding sources). Describe any assumptions made about any missing or unclear information. | 7-8 |
| Study risk of bias assessment | 11 | Specify the methods used to assess risk of bias in the included studies, including details of the tool(s) used, how many reviewers assessed each study and whether they worked independently, and if applicable, details of automation tools used in the process. | 8 |
| Effect measures | 12 | Specify for each outcome the effect measure(s) (e.g. risk ratio, mean difference) used in the synthesis or presentation of results. | 8 |
| Synthesis methods | 13a | Describe the processes used to decide which studies were eligible for each synthesis (e.g. tabulating the study intervention characteristics and comparing against the planned groups for each synthesis (item #5)). | 7 |
|  | 13b | Describe any methods required to prepare the data for presentation or synthesis, such as handling of missing summary statistics, or data conversions. | 8 |
|  | 13c | Describe any methods used to tabulate or visually display results of individual studies and syntheses. | 8 |
|  | 13d | Describe any methods used to synthesize results and provide a rationale for the choice(s). If meta-analysis was performed, describe the model(s), method(s) to identify the presence and extent of statistical heterogeneity, and software package(s) used. | 8 |
|  | 13e | Describe any methods used to explore possible causes of heterogeneity among study results (e.g. subgroup analysis, meta-regression). | 8 |
|  | 13f | Describe any sensitivity analyses conducted to assess robustness of the synthesized results. | 11 |
| Reporting bias assessment | 14 | Describe any methods used to assess risk of bias due to missing results in a synthesis (arising from reporting biases). | 8 |
| Certainty assessment | 15 | Describe any methods used to assess certainty (or confidence) in the body of evidence for an outcome. | 8 |
| **RESULTS** | | |  |
| Study selection | 16a | Describe the results of the search and selection process, from the number of records identified in the search to the number of studies included in the review, ideally using a flow diagram. | 8 |
|  | 16b | Cite studies that might appear to meet the inclusion criteria, but which were excluded, and explain why they were excluded. | Figure 1 (Flow diagram) |
| Study characteristics | 17 | Cite each included study and present its characteristics. | 8-9 (Tables 1A/1B) |
| Risk of bias in studies | 18 | Present assessments of risk of bias for each included study. | 9 (Table 2) |
| Results of individual studies | 19 | For all outcomes, present, for each study: (a) summary statistics for each group (where appropriate) and (b) an effect estimate and its precision (e.g. confidence/credible interval), ideally using structured tables or plots. | 9-11 (Forest Plots) |
| Results of syntheses | 20a | For each synthesis, briefly summarise the characteristics and risk of bias among contributing studies. | 9 |
|  | 20b | Present results of all statistical syntheses conducted. If meta-analysis was done, present for each the summary estimate and its precision (e.g. confidence/credible interval) and measures of statistical heterogeneity. If comparing groups, describe the direction of the effect. | 9-11 |
|  | 20c | Present results of all investigations of possible causes of heterogeneity among study results. | 9-11 |
|  | 20d | Present results of all sensitivity analyses conducted to assess the robustness of the synthesized results. | 9-11 |
| Reporting biases | 21 | Present assessments of risk of bias due to missing results (arising from reporting biases) for each synthesis assessed. | 11 |
| Certainty of evidence | 22 | Present assessments of certainty (or confidence) in the body of evidence for each outcome assessed. | 11 (Table 3) |
| **DISCUSSION** | | |  |
| Discussion | 23a | Provide a general interpretation of the results in the context of other evidence. | 11-12 |
|  | 23b | Discuss any limitations of the evidence included in the review. | 14 |
|  | 23c | Discuss any limitations of the review processes used. | 14 |
|  | 23d | Discuss implications of the results for practice, policy, and future research. | 14 |
| **OTHER INFORMATION** | | |  |
| Registration and protocol | 24a | Provide registration information for the review, including register name and registration number, or state that the review was not registered. | 5 |
|  | 24b | Indicate where the review protocol can be accessed, or state that a protocol was not prepared. | 5 |
|  | 24c | Describe and explain any amendments to information provided at registration or in the protocol. | 5 |
| Support | 25 | Describe sources of financial or non-financial support for the review, and the role of the funders or sponsors in the review. | 2 |
| Competing interests | 26 | Declare any competing interests of review authors. | 26 |
| Availability of data, code and other materials | 27 | Report which of the following are publicly available and where they can be found: template data collection forms; data extracted from included studies; data used for all analyses; analytic code; any other materials used in the review. | 2 |

###

###

###

###

###

### **Supplementary Table 2. Full Search Strategies**

| **1. PubMed (Searched October 16, 2025)** | "Dexmedetomidine"[Mesh] OR dexmedetomidine[tiab] )  AND  ( "Tachycardia, Ectopic Junctional"[Mesh] OR "Arrhythmias, Cardiac"[Mesh] OR "junctional ectopic tachycardia"[tiab] OR JET[tiab] OR arrhythmia*[tiab] )  AND  ( "Pediatrics"[Mesh] OR "Child"[Mesh] OR "Infant"[Mesh] OR "Cardiac Surgical Procedures"[Mesh] OR "Cardiopulmonary Bypass"[Mesh] OR "Heart Defects, Congenital"[Mesh] OR pediatric*[tiab] OR paediatric*[tiab] OR child*[tiab] OR infant*[tiab] OR "congenital heart surgery"[tiab] OR "cardiopulmonary bypass"[tiab] ) |
| --- | --- |
| **2. Scopus (Searched October 16, 2025)** | ( TITLE-ABS-KEY ( "dexmedetomidine" ) )  AND  ( TITLE-ABS-KEY ( "Tachycardia, Ectopic Junctional" OR "Arrhythmias, Cardiac" OR "junctional ectopic tachycardia" OR JET OR arrhythmia* ) )  AND  ( TITLE-ABS-KEY ( Pediatrics OR Child OR Infant OR "Cardiac Surgical Procedures" OR "Cardiopulmonary Bypass" OR "Heart Defects, Congenital" OR pediatric* OR paediatric* OR child* OR infant* OR "congenital heart surgery" OR "cardiopulmonary bypass" ) ) |
| **3. Cochrane Central Register of Controlled Trials (CENTRAL) [Searched October 16, 2025]** | (dexmedetomidine)  AND  ("Tachycardia, Ectopic Junctional" OR "Arrhythmias, Cardiac" OR "junctional ectopic tachycardia" OR JET OR arrhythmia*)  AND  (Pediatrics OR Child OR Infant OR "Cardiac Surgical Procedures" OR "Cardiopulmonary Bypass" OR "Heart Defects, Congenital" OR pediatric* OR paediatric* OR child* OR infant* OR "congenital heart surgery" OR "cardiopulmonary bypass") |

#

# **Supplementary Table 3 – Excluded Studies and Reasons**

| **Study (Author, Year)** | **Reason for Exclusion** |
| --- | --- |
| **Shuplock et al., 2014** | Retrospective cohort; no randomized/control arm and JET outcome not extractable. |
| **Chrysostomou et al., 2010** | Prospective observational ECG study; no prophylactic intervention; no JET incidence reported. |
| **Chrysostomou et al., 2011** | Prospective cohort; not randomized; mixed arrhythmia outcomes, not JET-specific. |
| **Gautam et al., 2017** | Quasi-randomized allocation; non-comparable single-lesion population; key outcomes (VIS, ventilation, ICU stay) not extractable; high clinical and methodological heterogeneity. |
| **El-Shmaa et al., 2016** | Dex vs amiodarone only; lacks placebo/control group; effect vs standard care cannot be isolated. |
| **Zhou et al., 2024** | Outcome not JET-specific; evaluated general arrhythmias (SVT/VT). |
| **Nagao et al., 2022** | Case report; wrong design; therapeutic use, not prophylaxis. |
| **Ortmann et al., 2019** | Retrospective cohort; dex used for sedation, not prophylaxis; arrhythmias not JET-specific. |
| **Chrysostomou et al., 2013** | Retrospective case series on SVT termination; wrong arrhythmia and therapeutic use. |
| **Chrysostomou, 2012** | Editorial paper; no original data; cannot extract outcomes. |
| **Chrysostomou et al., 2011 (Thorac Surg)** | Prospective cohort; non-randomized; includes mixed arrhythmias, not strictly JET. |
| **Chrysostomou et al., 2006** | Retrospective case series on postoperative sedation; no prophylaxis; wrong outcomes. |
| **Chrysostomou et al., Intensive Care Med 2010** | Observational ECG study; no JET outcomes and not prophylaxis. |
| **El Amrousy et al., 2016** | Three-arm RCT without true standard-care control; comparator violates PICOS. |
| **Misc SVT/EAT therapeutic studies** | Wrong arrhythmia subtype (SVT/EAT) and therapeutic use, not JET prophylaxis. |

###

###

###

###

###

###

###

###

###

###

###

###

###

###

###

###

###

###

###

###

###

**Supplementary Table 4. GRADE Summary of Findings for Prophylactic Dexmedetomidine**

| **Outcome** | **Effect Estimate (95% CI)** | **No. of Studies** | **Study Design** | **Risk of Bias** | **Inconsistency** | **Indirectness** | **Imprecision** | **Publication Bias** | **Overall Certainty** |
| --- | --- | --- | --- | --- | --- | --- | --- | --- | --- |
| **Incidence of JET** | **OR 0.37** [0.23, 0.58] | 5 | RCTs | Serious  (-1) | Not Serious(0) | Not Serious(0) | Not Serious(0) | Not Suspected (0) | Moderate⊕⊕⊕◯ |
| **Vasoactive-Inotropic Score (VIS)** | **MD -1.15** [-2.66, 0.36] | 4 | RCTs | Serious  (-1) | Very Serious(-2) | Not Serious(0) | Serious (-1) | Not Suspected (0) | Very Low  ⊕◯◯◯ |
| **Ventilation Time** | ​​**MD -4.80**  [-9.43, -0.18] | 4 | RCTs | Serious  (-1) | Very Serious(-2) | Not Serious(0) | Serious (-1) | Not Suspected (0) | Very Low  ⊕◯◯◯ |
| **ICU Stay** | **MD -19.83** [-36.84, -2.83] | 5 | RCTs | Serious  (-1) | Very Serious(-2) | Not Serious(0) | Serious (-1) | Not Suspected (0) | Very Low  ⊕◯◯◯ |
| **Mortality** | **OR 0.37** [0.11 to 1.25] | 5 | RCTs | Serious  (-1) | Not Serious(0) | Not Serious(0) | Very Serious (-2) | Not Suspected (0) | Low  ⊕⊕◯◯ |
| **Hypotension** | **OR 1.07** [0.31 to 3.67] | 3 | RCTs | Serious (-1) | Not Serious(0) | Not Serious(0) | Very Serious (-2) | Not Suspected (0) | Low  ⊕⊕◯◯ |

**Abbreviations:** CI, Confidence Interval; MD, Mean Difference; OR, Odds Ratio; RCT, Randomized Controlled Trial; JET, Junctional Ectopic Tachycardia.

**Footnotes:** (a) **Risk of Bias (Serious):** Certainty downgraded due to high risk of bias in multiple trials using non-random or "quasi-random" allocation methods (e.g., "alternate sampling," "every second patient," "drawing of a chit"), introducing a high risk of selection bias. (b) **Inconsistency (Very Serious):** Certainty downgraded due to very high statistical heterogeneity (I² > 80%), indicating significant disagreement in results across studies. (c) **Imprecision (Serious):** Certainty downgraded as the confidence interval was borderline or crossed the line of no effect (VIS, Ventilation Time) or was too wide to be clinically precise (ICU Stay). (d) **Imprecision (Very Serious):** Certainty downgraded due to a very low number of total events (Mortality: 14 events; Hypotension: 11 events), making the effect estimate unstable and resulting in a very wide confidence interval that crosses the line of no effect.

**Supplementary Figure S1: Traffic Light Plot**


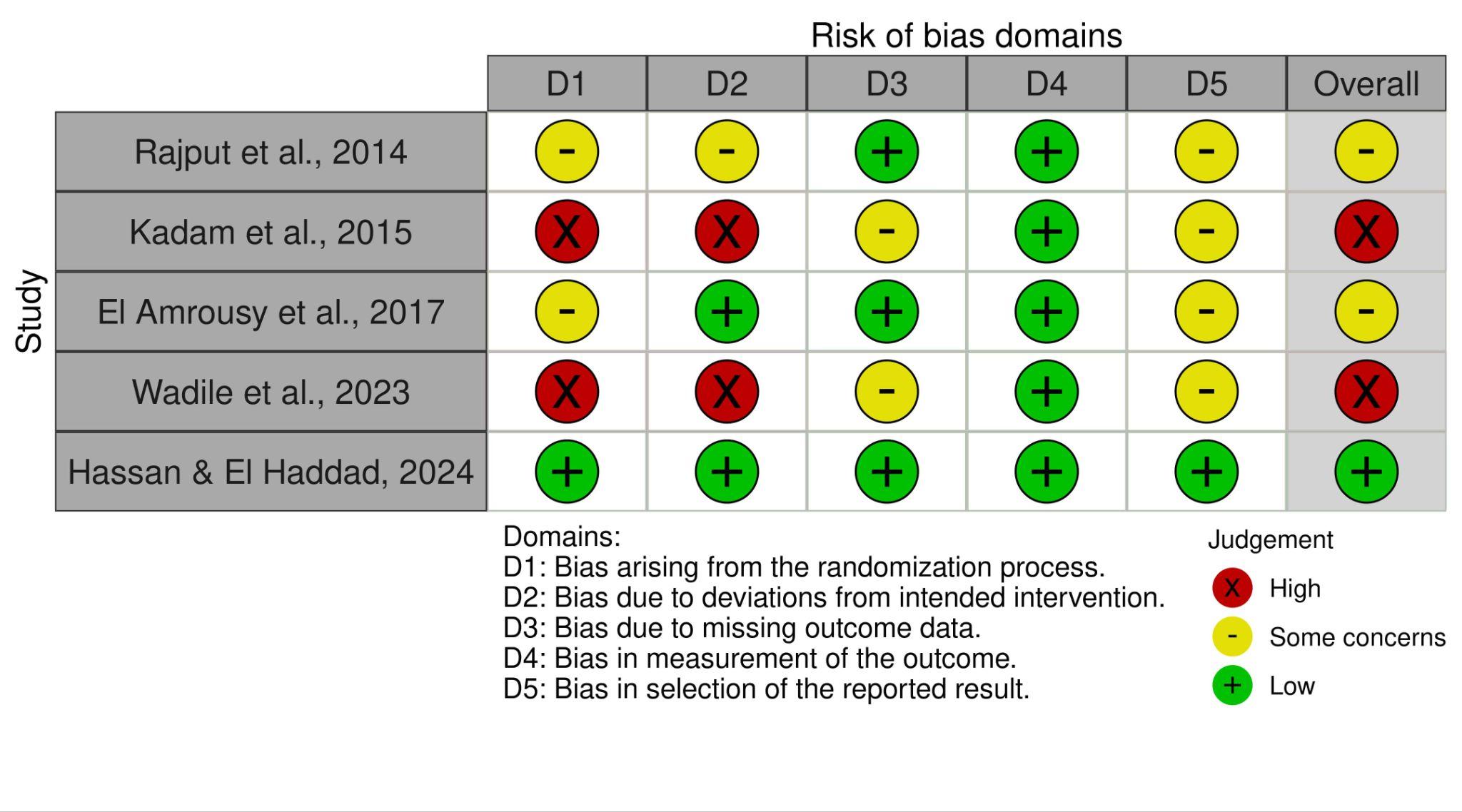


### **Supplementary Figure S2. Sensitivity Analysis for Incidence of JET**

***
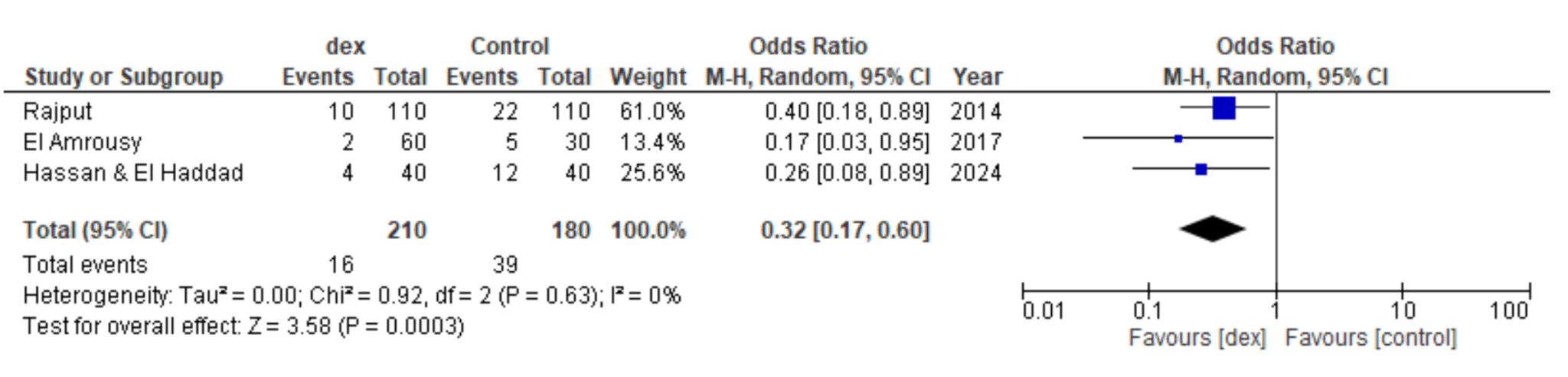
***

**Figure S2.** Sensitivity analysis for the primary outcome (Incidence of JET), excluding studies with a high risk of bias (Kadam et al., Wadile et al.).

### **Supplementary Figure S3. Sensitivity Analysis for Vasoactive-Inotropic Score (VIS)**

***
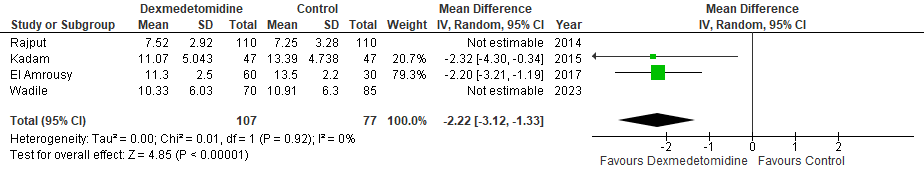
***

**Figure S3.** Sensitivity analysis for Vasoactive-Inotropic Score (VIS), excluding studies with heterogeneous calculation methods (Rajput et al., 2014; Wadile et al., 2023).

### **Supplementary Figure S4. Sensitivity Analysis for Ventilation Time**

***
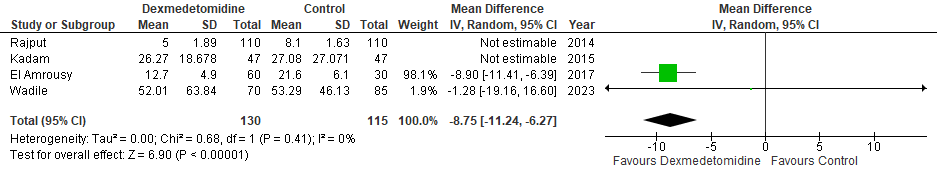
***

**Figure S4**. Sensitivity analysis for Ventilation Time, excluding studies with clinically heterogeneous populations (TOF-only cohorts) (Rajput et al., 2014; Kadam et al., 2015).

**Supplementary Figure S5. Sensitivity Analysis for ICU Stay**

***
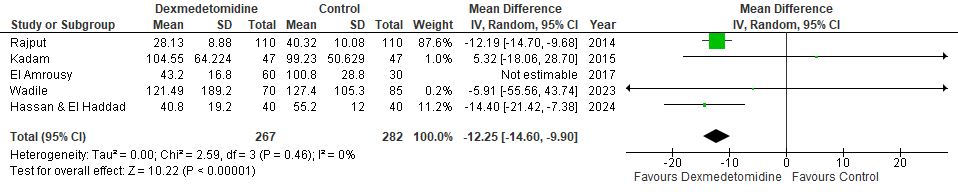
***

**Figure S5.** Sensitivity analysis for ICU Stay, excluding statistical outlier (El Amrousy et al., 2017).
